# Lipid droplets and ferroptosis as new players in brain cancer glioblastoma progression and therapeutic resistance

**DOI:** 10.3389/fonc.2022.1085034

**Published:** 2022-12-15

**Authors:** Ayenachew Bezawork-Geleta, James Dimou, Matthew J. Watt

**Affiliations:** ^1^ Department of Anatomy and Physiology, School of Biomedical Sciences, The University of Melbourne, Melbourne, VIC, Australia; ^2^ Department of Surgery, The University of Melbourne, Parkville, VIC, Australia; ^3^ Department of Neurosurgery, The Royal Melbourne Hospital, Parkville, VIC, Australia

**Keywords:** brain cancer, metabolism, lipids, cell death, therapeutic vulnerabilities, glioma, lipid droplet (LD)

## Abstract

A primary brain tumor glioblastoma is the most lethal of all cancers and remains an extremely challenging disease. Apparent oncogenic signaling in glioblastoma is genetically complex and raised at any stage of the disease’s progression. Many clinical trials have shown that anticancer drugs for any specific oncogene aberrantly expressed in glioblastoma show very limited activity. Recent discoveries have highlighted that alterations in tumor metabolism also contribute to disease progression and resistance to current therapeutics for glioblastoma, implicating an alternative avenue to improve outcomes in glioblastoma patients. The roles of glucose, glutamine and tryptophan metabolism in glioblastoma pathogenesis have previously been described. This article provides an overview of the metabolic network and regulatory changes associated with lipid droplets that suppress ferroptosis. Ferroptosis is a newly discovered type of nonapoptotic programmed cell death induced by excessive lipid peroxidation. Although few studies have focused on potential correlations between tumor progression and lipid droplet abundance, there has recently been increasing interest in identifying key players in lipid droplet biology that suppress ferroptosis and whether these dependencies can be effectively exploited in cancer treatment. This article discusses how lipid droplet metabolism, including lipid synthesis, storage, and use modulates ferroptosis sensitivity or tolerance in different cancer models, focusing on glioblastoma.

## Introduction

Glioblastoma, designated as WHO grade 4, is the most common and most aggressive intra-axial brain tumor and has limited treatment options (1–3). The development of cancer therapies has improved outcomes for most malignancies, with the five-year survival of patients ranging from 25–95% (4–6). In contrast, glioblastoma remains overwhelmingly lethal, with only ~5% of patients alive five years after diagnosis (7).

The gold standard treatment of glioblastoma includes maximal safe surgical resection (8) combined with radiotherapy and temozolomide chemotherapy, which is referred to in neuro-oncology circles as the Stupp protocol (1, 2)**;** however, relapse remains inevitable. Temozolomide (chemical name 3-methyl-4-oxoimidazo[5,1-d](1–3, 5)tetrazine-8-carboxamide (9)) has several advantages, such as oral administration, a favorable side-effect profile, evidence of blood–brain barrier penetration, acidic environment stability, and limited drug interaction-related toxicity. The original randomized controlled trial published by Stupp et al. (1, 2) showed that adding postoperative adjuvant temozolomide chemotherapy to surgery and radiotherapy increased the median survival of glioblastoma patients from 12.1 months to 14.6 months. Bevacizumab, a monoclonal antibody targeted to vascular endothelial growth factor A (VEGF-A), has been shown to offer some progression-free survival benefits in patients who develop recurrent glioblastoma (10). Another study has shown that epigenetic silencing of the DNA repair gene O-6-methylguanine-DNA methyltransferase (MGMT) offers a superior response to chemoresistance tumors to temozolomide (11). However, it has limited therapeutic efficacy due to recurrence.

For three decades, other strategies have been pursued to improve the survival outcomes of glioblastoma patients (12, 13). Considerable efforts have been made to catalog genetic aberrations and associated disrupted signaling pathways in glioblastoma for the purpose of developing novel targeted therapies. The first genome atlas study by The Cancer Genome Atlas (TCGA) Research Network uncovered 453 validated missense somatic mutations in glioblastoma (14). Subsequent systematic analysis at the genomic and transcriptomic levels showed that 71 mutated genes as significant pathogenic factors (14, 15). Histological and molecular analysis further revealed a landscape of tumor heterogeneity, which led to efforts to differentiate glioblastoma into distinct molecular subtypes (16, 17). An early attempt at such molecular classification by Verhaak et al. distinguished glioblastoma into four molecular subtypes: proneural, mesenchymal, classical, and neural (18). The classical subtype was characterized by amplification of epidermal growth factor receptor (EGFR) and associated with the upregulation of retinoblastoma (RB), sonic hedgehog (SHH), and notch signaling-related pathway genes. The classical type typically lacks of TP53 mutation. Conversely, mesenchymal subtype glioblastomas were characterized by high expression of chitinase 3 like 1 (CHI3L1) and tyrosine-protein kinase Met (MET), a high frequency of neurofibromin 1 (NF1) mutation/deletion, and low NF1 gene expression. Key proneural subtype markers are TP53 aberrations and metabolic enzyme isocitrate dehydrogenase 1 (IDH1) mutation and platelet-derived growth factor receptor alpha (PDGFRA). The neural subtype expressed neurofilament light chain (NEFL), gamma-aminobutyric acid type A receptor subunit alpha1 (GABRA1), synaptotagmin 1 (SYT1), and solute carrier family 12 member 5 (SLC12A5). It has been proposed that these specific subtypes of glioblastoma develop due to promutagenic aberrations in distinct cells of origin (18). However, due to the inclusion of mRNA from glioblastoma-associated stroma (nonmalignant cells) along with tumor cells in the Verhaak et al. transcriptome study, these four pathological subtypes subjected to further interrogation. Accordingly, Wang et al. (19) used stringent criteria to distinguish specific mRNA from peripheral nonmalignant cells by comparing the transcriptome of core versus peripheral glioblastoma surgery specimens and mRNA profile from glioblastoma cell culture and revealed the presence of only three pathological subtypes (i.e., classical, proneural, and mesenschymal subtypes). Subsequent studies also classify glioblastoma into different prognostic subtypes (20–22) using different multi-omics signatures.

Recently, the integration of cross-platform analyses coupling metabolomic profiling with genomics and proteomics has provided an in-depth understanding of the metabolic programming that occurs during tumor growth. It has identified key metabolic nodes specific to glioblastoma and their molecular context. Notable findings of mutations in the metabolic enzyme IDH1, representing an early event in gliomagenesis (22, 23), have led to an in-depth study of metabolic status across all grades and subtypes of glioma. However, it must be stressed that the presence of IDH1 mutation now precludes the formal pathological diagnosis of glioblastoma, as it is now genetically defined as “IDH-wildtype” in the most recent edition of the WHO Classification of CNS Tumors (24).

Further, it has been found that glucose, glutamine, and tryptophan metabolism play roles in glioblastoma progression and recurrence following surgery and chemotherapy (25–28). Glutamine metabolism changes in glioblastoma have been incorporated into the clinical practice of noninvasive metabolic imaging strategies for stratifying patients, monitoring treatment response, and prognostication (29–32). Advances in metabolic imaging modalities, including magnetic resonance spectroscopy (MRS), positron emission tomography (PET), single-photon emission computerized tomography (SPECT), mass spectrometry imaging (MSI), and fluorescence imaging have given researchers and clinicians unprecedented opportunities for *in vivo* measurements of glutamine metabolism and clinical management of gliomas (29–32). In particular, PET using ^11^C-glutamine allows noninvasive visualization of glioma and various malignant tumors in multiple organs and for subsequent monitoring of responses to clinical treatments (29).

Unfortunately, our ever-improving understanding of the molecular basis of glioblastoma initiation and progression has not yet translated to therapeutic success (17). This requires the interrogation of novel molecular signaling mechanisms and metabolic processes in glioblastoma and their exploitation for therapeutic progress. To this end, lipid metabolism and ferroptosis have only recently been explored and identified as key regulators in the initiation and maintenance of glioblastoma (33–35) and other cancers (36–38). Notable related discoveries and translations include the recently developed and characterized selective, irreversible, and potent fatty acid synthase (FASN) inhibitor IPI-9119 (39), as well as the identification of druggable targets, including ATP-citrate lyase (ACLY) (40) and the plasma membrane lipid importer CD36 (41). This review examines recent studies showing lipid droplets’ critical role in suppressing ferroptosis to promote tumorigenesis.

## Features of ferroptosis

Ferroptosis is a recently discovered type of programmed cell death that exhibits unique morphological, biochemical and genetic features compared to previously characterized processes of cell death, including apoptosis, necrosis, and autophagy (42–44). Accordingly, ferroptosis does not depend on the mechanisms by which cancer cells frequently evade apoptosis, such as the activation of caspases. Apoptosis inhibitors (e.g., Z-VAD-FMK, BOC-D-FMK, wortmannin, and necrostatin-1) failed to protect cells from ferroptosis mediated cell death (42–44). Instead, ferroptosis is mainly embodied by accumulation iron, leading to lethal levels of lipid peroxidation. The typical cells in ferroptosis show extensive lipid peroxidative damage to biological membranes, shrunken mitochondria, and an increase in mitochondrial membrane density (45), indicating that execution of ferroptosis requires active mitochondrial function. Interestingly, the nucleus remains intact during ferroptosis, in contrast to other cell death mechanisms that lead to fragmented nuclei (43, 46, 47). Ferroptosis is also caused by a redox imbalance of cellular homeostasis, leading to the formation of a myriad of secondary byproducts, including breakdown products of lipid peroxides (e.g., malondialdehyde (MDA), 4-hydroxynonenal (4HNE), 4-hydroxyhexenal (4HHE), and 4-oxo-nonenal (4ONE)) and oxidized and modified proteins). This chain reaction may eventually lead to the breakdown of membrane integrity and the rupture of organelles and/or cell membranes and, ultimately, result in cell death (43–45, 48). In contrast, ferroptosis-mediated cell death can be modulated by the expression and activity of proteins that regulate the levels, transport, storage, and metabolism of iron, cystine, cysteine, GSH, glutamine, and selenium (43–45, 48). Antioxidant defense enzymes that repair oxidative damage to lipids, such as Glutathione peroxidase 4 (GPX4), are important inhibitors of ferroptosis (43–45, 48). Recently, it has been reported that Anti-TfR1 3B8 2A1 and anti-MDA 1F83 can be used to detect ferroptosis in both cell culture and xenograft tumor sections (49).

Although it was initially proposed to be the mechanism of neuronal death in many neurodegenerative diseases (43, 50, 51), emerging data indicate that ferroptosis plays an important role in cancer development and drug resistance (45). Therefore, inducing ferroptosis represents a potentially orthogonal approach to drug discovery to kill cancer cells that have developed resistance to apoptosis. Thus, a clearer understanding of ferroptosis in glioblastoma and its role in drug resistance may positively impact clinical practice (52, 53).

## In and out of lipids: Lipid droplets as intracellular sources of fatty acids

### Lipid droplets accumulate in cancers

Lipid droplets are fundamental in the storage and release of fatty acids. Lipid droplets are ubiquitously present organelles in many cells with a hydrophobic core surrounded by a single layer of phospholipids decorated with various sets of proteins. The hydrophobic core contains neutral lipids, including triacylglycerols, sterol esters, retinyl esters and 1-O-acylceramides (54–57). Emerging evidence indicated lipid droplet accumulation in several cancers (58, 59), especially in hypoxic cancer cells, and several studies have linked lipid droplet abundance with more aggressive tumor phenotype (34, 36, 58, 60). It has also shown lipid metabolism-dependent proliferation and survival of glioblastoma following radiation (61, 62), antiangiogenic (63) and ketone diet therapy (64). As such, they have recently been proposed to play direct roles in many cancers and considered a hallmark of fundamental tumor processes (58, 60, 65). The following sections discuss the possible roles of lipid droplet metabolism, including (1) fatty acid storage as neutral lipids, (2) lipolysis of neutral lipids, and (3) lipophagy in limiting or triggering ferroptosis.

### *De novo* lipogenesis and ferroptosis

**Figure 1:**
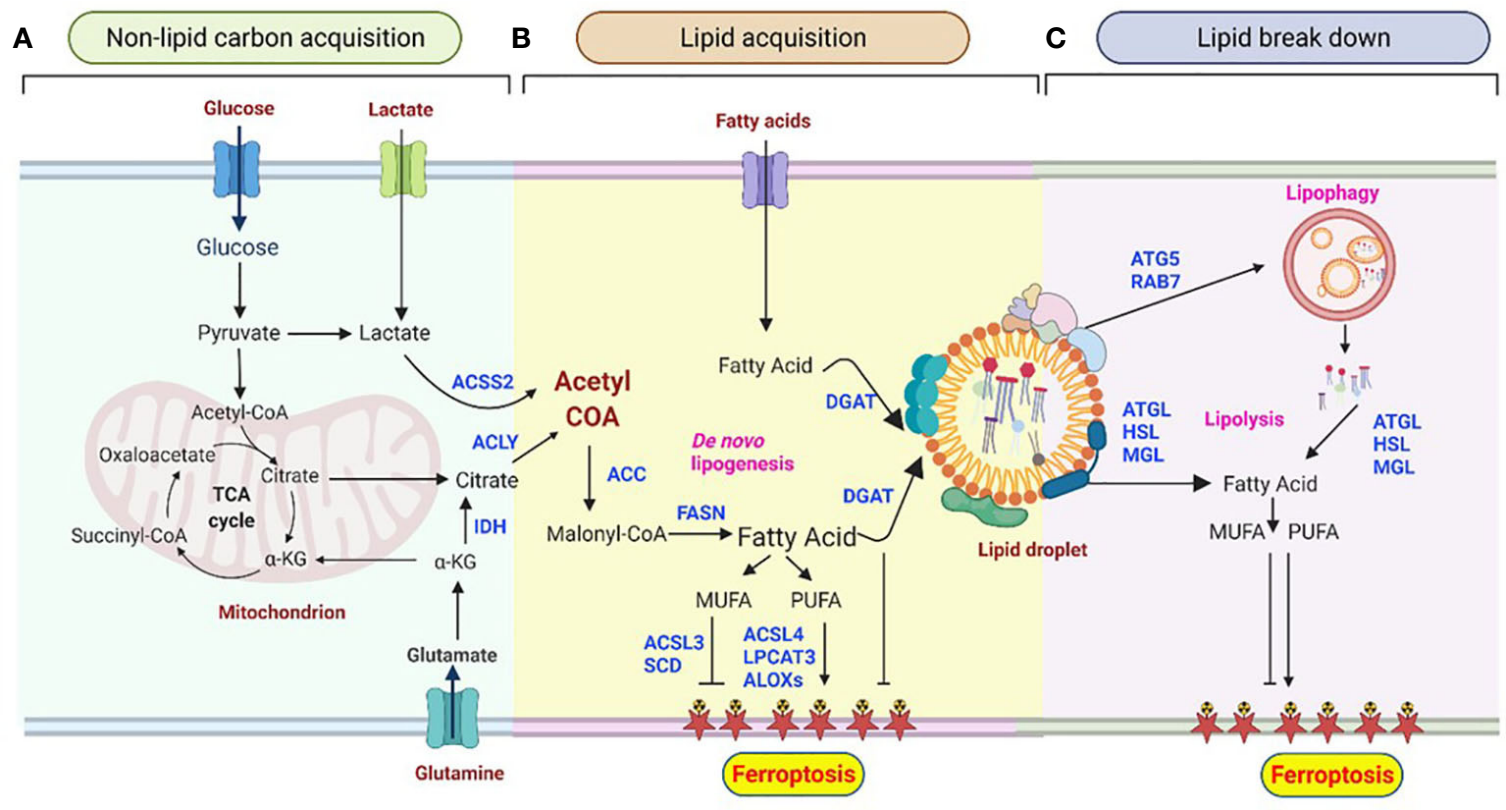
Lipid droplet metabolism is central to shaping the ferroptotic response. **(A)** Non-lipid carbon acquisition of carbon in cancer cells. Glucose- and glutamine-derived citrate, which results from increased glycolysis and glutaminolysis, is first converted to acetyl-coenzyme A (acetyl-CoA) by ATP-citrate lyase (ACLY). Acetyl-CoA can also be derived from acetate. **(B)** *De novo* lipogenesis (DNL) and esterification in ferroptosis. Acetyl-CoA is converted to malonyl-CoA by acetyl-CoA carboxylase (ACC) and condensed by fatty acid synthase (FAS). Acyl-CoA synthetase long-chain 4 (ACSL4) and lysophosphatidylcholine acyltransferase 3 (LPCAT3) mediate the production of polyunsaturated fatty acids (PUFAs), which are essential for the induction of ferroptosis. In contrast, acyl-CoA synthetase long-chain 3 (ACSL3) and stearoyl CoA desaturase (SCD) contribute to the synthesis of monounsaturated fatty acids (MUFAs), leading to ferroptosis resistance. Arachidonate lipoxygenases (ALOXs) catalyze the stereospecific insertion of oxygen into PUFAs, thereby promoting ferroptosis. Diacylglycerol acyltransferase (DGAT1/2)-mediated triglyceride synthesis and lipid droplet formation act as a sink for free fatty acids, thus preventing their peroxidation.**(C)** Lipid degradation in ferroptosis. The selective degradation of lipid droplets by member RAS oncogene family 7 (RAB7A)- and adipocyte triglyceride lipase (ATGL)-related lipophagy increases the production of free fatty acids for subsequent ferroptosis. Lipolysis may provide PUFAs, thus stimulating lipid peroxidation and sensitizing cells to ferroptosis. Lipases may also provide MUFAs that reduce the abundance of oxidizable PUFAs in membranes, thereby restricting lipid peroxidation and ferroptosis. Lipid droplets act as buffers of lipid flux and release, thereby emerging as master regulators of ferroptotic sensitivity.

Lipogenesis (lipid acquisition) is an energy-expensive process of fatty acid synthesis and subsequent esterification to neutral lipids into lipid droplets. The primary process of synthesizing fatty acids from acetyl‐CoA subunits that are produced most commonly from carbohydrate catabolism is called *de novo* lipogenesis (DNL) (66). Acetyl-coenzyme A (acetyl-CoA) is a key metabolite precursor in DNL, and its abundance is closely monitored by cellular adaptive mechanisms. While glucose is the most common supplier of carbon units for DNL (**Figure 1A**), fructose is metabolized to glycerol and subsequently converted to a lipogenic substrate glycerol-3-phosphate (G3P) that can drive DNL.

**Figure 2:**
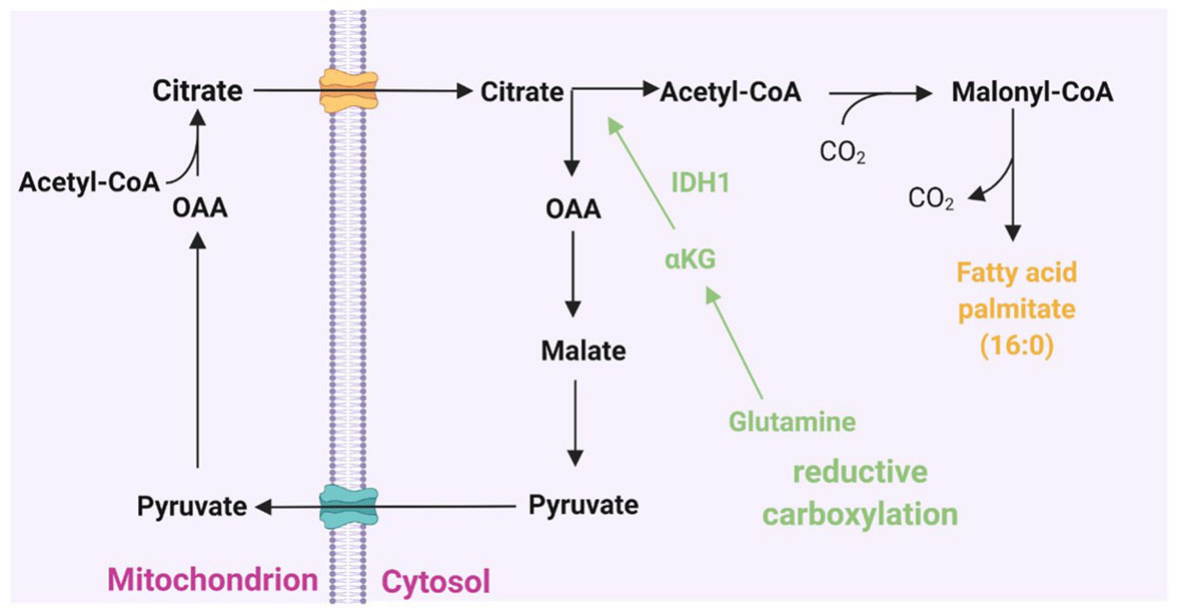
Cytoplasmic citrate pool and fatty acid synthesis. Acetyl-CoA couples with oxaloacetate (OAA) to form citrate at the beginning of the citric acid cycle. Citrate can then shuttle across the mitochondrial membrane to the cytoplasmic citrate pool. In the cytosol, citrate lyase splits citrate back into acetyl-CoA and OAA. The latter can then return to the mitochondrion. Acetyl-CoA is activated in the cytoplasm for incorporation into fatty acids by acetyl-CoA carboxylase to form malonyl-CoA and then to *de novo* fatty acid synthesis. Glutamine-derived α-ketoglutarate (α-KG) can add to the cytoplasmic citrate pool *via* reductive carboxylation to provide an alternative source of lipid synthesis.

Glutamine is another non-lipid metabolite that contributes carbon to lipogenic acetyl-CoA (**Figure 1A**) through two distinct pathways: Glutamine metabolism can produce citrate either through the tricarboxylic acid (TCA) cycle in mitochondria or by the reductive carboxylation of cytoplasmic α-ketoglutarate (αKG) to citrate by IDH1 (**Figure 2**) (67, 68). Interestingly, many cancer cells, including glioblastoma, generate 10–25% of their lipogenic acetyl-CoA from glutamine through reductively carboxylation (69), highlighting the general use of reductive carboxylation as the primary route to convert non-lipid carbon to lipids in cancer cells. In agreement with this, blood-borne glucose contribute minimal fraction to the acetyl-CoA pool of glioblastoma and brain metastases that originate from other tissues (70). In addition to a direct contribution of supplying a carbon unit, glucose and glutamine uptake promotes lipogenesis through transcriptional regulation in a sterol regulatory element-binding protein (SREBP) dependent manner in glioblastoma (71, 72).

Acetate, often dispensable for cells, has also been shown as a source of acetyl-CoA for DNL in cancer cells, especially primary glioblastomas and other cancer metastasis to brain (73, 74). In this case, three important processes need to be considered: acetate production, its intercellular import across the cell membrane, and the carbon fluxes from acetate to lipids. Imported acetate is converted to acetyl-CoA in a reaction catalyzed by acetyl-CoA synthetase 2 (ACSS2) (**Figure 1A**) (75). Thus, hypoxia-associated deficits in acetyl-CoA can be supplemented by increasing the use of free extracellular acetate (including plasma, interstitial fluid, and neighboring cells) and the intercellular pool of mainly acetate released from the deacetylation of histones and other cellular proteins (76). In line with acetate metabolism, apparent ACSS2 expression is associated with poor progenesis in glioblastoma patients (73, 74). Despite the fundamental importance of acetate and its vital roles to several intracellular and extracellular metabolism, little is known about the mechanisms and regulatory processes in acetate metabolism.

Cytoplasmic acetyl-CoA is then carboxylated by acetyl-CoA carboxylase (ACC) to produce malonyl-CoA (**Figure 1B**), which is further used to synthesizes medium-and long-chain fatty acids. The key three proteins involved in the initial fatty acyl chain biosynthesis, ACLY, ACC, and FASN, are commonly upregulated and activated in many cancers (36, 60, 65), and they are in the frontline of pre-clinical target evaluations, drug discovery pipelines, and clinical trials. For instance, the therapeutic targeting of FASN is currently being explored in a phase 2 clinical trial in patients with glioblastoma using the FASN inhibitor TVB-2640 in combination with bevacizumab (NCT03032484) (28).

*De novo* synthesized fatty acids are then further esterified to neutral lipids and stored in their nontoxic (inert, neutral) form in lipid droplets (**Figure 1B**). Esterification starts with the acylation of glycerol-3-phosphate with two fatty acids to produce phosphatidic acid (PA), followed by dephosphorylation to yields diacylglycerol (DAG). DAG then serves as a precursor for the synthesis of both phospholipids and acylation to yield triacylglycerols (TAGs), which is catalyzed by diacylglycerol acyltransferase 1 and 2 (DGAT1 and DGAT2) (77, 78). TAG is stored in lipid droplets as energy reservoir, supplying the cell with energy when required.

Lipid droplets also store 1-O-acylceramides and cholesteryl ester. Although some studies indicated a minimal abundance of1-O-acylceramides in tissue (0.25-1.3 per mg of mice tissue), the indication of its level during Western diet consumption (79) warrants special attention, mainly its correlation with cancer progression in obese glioblastoma patients. Cholesteryl ester is another neutral lipid stored in lipid droplets. Acyl-CoA acyltransferase, also known as acyl-Coenzyme A or cholesterol acyltransferase (SOAT1 and SOAT2), catalyzes the formation of cholesteryl esters, using cholesterol and long-chain fatty acyl-CoA as substrates. Both isoforms of acyl-CoA acyltransferase (ACAT1 and ACAT2) are highly expressed and post-translationally regulated in glioblastomas and other cancers, and their expression and activation levels correlate with patient survival (79–81). *In vivo* studies showed that both genetic silencing of ACAT1 or blocking its activity using inhibitors suppresses tumor growth (80, 82, 83). These suggest that targeting ACAT1 and cholesteryl ester synthesis may be a promising anticancer strategy.

The *de novo* synthesis of fatty acids can be extended to the formation of either saturated or unsaturated fatty acids. Unsaturated fatty acids can be further classified into monounsaturated fatty acids [MUFAs, with only one double bond, e.g., oleic acid (18:1)] and polyunsaturated fatty acids [PUFAs, with at least two double bonds, e.g., linoleic acid (18:2 n-6) and α-linolenic (18:3 n-3)]. Unsaturated fatty acids can bind membrane phospholipids in differential degrees that subject tumor cells to oxidative stress. Studies have revealed that an increase in the MUFA/PUFA ratio results in fewer peroxidation-susceptible targets, ultimately reducing the susceptibility of cancer cells to ferroptosis (84). On the other hand, an increase in the synthesis of PUFAs can promote subsequent lipid peroxidation in cancer cells under oxidative stress conditions, suggesting that the formation of acetyl-CoA derivatives (*de novo* synthesis) of these PUFA is necessary for producing ferroptosis death signals (43, 45). Similarly, the esterification of fatty acids into neutral lipids has been shown to deplete the substrates for lipid peroxidation and increase the resistance of cancer cells to ferroptosis-mediated cell death (34, 36, 60, 85).

Lipid peroxidation normally occurs at a slow rate because PUFAs are required to react with the free radicals generated by iron acyl-CoA synthase long-chain family member 4 (ACSL4). Using genetic ablation and pharmaceutical inhibition, it has been shown that ACSL4 is a rate-limiting enzyme to catalyze the conversion of long-chain PUFAs, such as arachidonic acid and eicosapentaenoic acid to PUFA-CoA, and ultimately this increases lipid peroxidation and decreases resistance to ferroptosis (86–88). Under oxidative or energetic stress, PUFAs, particularly arachidonoyl (AA, 20:4 n-6) and adrenic acid (AdA), are oxygenated by different classes of enzymes, including ACSL4, lysophosphatidylcholine acyltransferase (LPCAT), and 15-lipoxygenase (15-LOX/ALOX15), to generate PUFA-containing phospholipids and various bioactive lipids that modulate sensitivity to ferroptosis. In this regard, genetic or pharmacological inhibition of ACSL4 suppresses ferroptosis in glioblastoma (86). On the other hand, overexpression of ACSL4 was shown to increase the levels of ferroptosis markers, including 5-hydroxyeicosatetraenoic acid (HETE), 12-HETE, and 15-HETE (86), indicating a key role of ACSL4 in regulating ferroptosis and proliferation of glioma cells (89). Independent experimental evidence showed that suppressing miR-670-3p, which targets ACSL4, also modulates the sensitivity of glioblastoma cells to ferroptosis-mediated cell death (87, 90).

### Esterification and ferroptosis

Blocking the esterification of fatty acyl-CoA prevents not only the formation of triglycerides but also modifies cell resistance to ferroptosis. It has been shown that in 3D tumor spheroids and *in vivo*, DGAT inhibition induces significant cytotoxic effects, especially when combined with dietary long-chain PUFAs (LC-PUFAs), implicating the combination of diet (LC-PUFAs) and DGAT inhibitor (DFATi) administration as a highly relevant therapeutic combination to induce ferroptosis (85). Mechanistically, DGATi administration inhibits lipid droplet formation resulting in the availability of more LC-PUFAs for peroxidation and ferroptotic cell death (85). In contrast, DGAT1 inhibition was recently reported to drive fatty acid-dependent oxidation in mitochondria generating high levels of reactive oxygen species, leading to apoptosis and significant growth inhibitory effects in glioblastoma (91). These contradictory reports are likely due to the difference in cell lineage, or a potential secondary effect associated with DGAT1 inhibition. Therefore, it will be relevant in the future to dissect the relative contributions of DGAT in different cancers, including glioblastoma, and the potential overlapping effects of DGAT inhibition on ferroptosis and apoptosis.

### Lipolysis and lipophagy and their role in cell sensitivity to ferroptosis

Similar to lipid fluxes into lipid droplets that act as buffers, the release of fatty acids from storage controls the cancer cells’ fate, including ferroptosis. Lipid droplet breakdown occurs *via* two major mechanisms-lipolysis, and lipophagy (**Figure 1C**), which provide a substrate for lipid peroxidation during ferroptosis. Unlike lipogenesis, the lipolysis process enables a highly regulated release of fatty acids from TAGs and is mediated by three essential lipases: adipocyte triglyceride lipase (ATGL), hormone-sensitive lipase (HSL), and monoacylglycerol lipase (MGL) (92–94). These three enzymes work together to promote the lipolysis of triglycerides and produce fatty acids and glycerol. ATGL catalyzes the first rate-limiting reaction whereby triglycerides are hydrolyzed to DAG. HSL then transforms DAG to monoacylglycerol (MAG), and MGL finally converts MAG to fatty acids and glycerol (93–96). However, the molecular regulation of the lipolysis of lipid droplet-containing triglycerides is complex. It involves a combination of subcellular localization, posttranslational modification (particularly phosphorylation), and protein-protein interactions (92, 97). For instance, hypoxia-induced lipid droplet-associated protein (HILPDA), also called hypoxia-induced gene-2 (HIG2), inhibits ATGL activity (98). Experimental evidence showed the correlation of the HIG2 expression with the glioma tumor grade and glioblastoma patient prognosis (34, 99).

Catabolism of lipid droplets also occur *via* lipophagy, a form of selective (macro)autophagy. Lipophagy is a process that tags specific lipid droplets and traffic whole lipid droplets to lysosomes for bulk degradation (**Figure 1C**). Lysosomal localized hydrolytic enzymes, such as triglycerides and cholesteryl ester hydrolase lysosomal acid lipase (LAL) (100), liberate neutral lipids stored in lipid droplet and generate free fatty acids and cholesterol in the cell. Although mechanistically lipolysis (the step-wise release of stored neutral lipids from lipid droplets) and lipophagy (a complete breakdown of lipid droplets) are distinct metabolic processes, it is not yet clear whether there is considerable crosstalk between the two processes and this may serve a distinct purpose in the cell (100, 101). It has been shown that perilipins (PLINs), lipid droplet surface proteins, act as gatekeepers to the lipophagy process and their degradation is a prerequisite for lipophagy. During starvation, perilipin 2 (PLIN2) and perilipin 3 (PLIN3) are proteolytically removed in parallel with the translocation of cytosolic ATGL and macroautophagy proteins onto lipid droplets (102). There is also evidence that PLIN2-mediated lipophagy regulation emerges as a key nodal point in modulating cellular sensitivity to ferroptosis. PLIN2 affects the proliferation of gastric carcinoma cells by modulating ferroptosis-related genes, including acyl-CoA synthetase long-chain family member 3 (ACL3), arachidonate 15-lipoxygenase (ALOXs), microtubule-associated protein 1 light chain 3 alpha (LC3), and the transcription factors pr/set domain 11 and importin 7 (IPO7) (103). Likewise, PLIN2 expression contributed to a decreased arachidonate 15-lipoxygenase (ALOX15) expression and arrested the occurrence of ferroptosis in gastric cancer. Conversely, PLIN2 knockdown facilitated a higher ALOX15 expression and accelerated ferroptosis (103). In contrast, increased lipid storage by tumor protein D52 (TPD52), a PLIN2 interactor protein, diminished lipid peroxidation to trigger ferroptosis (104). Recently, PLIN2 was also shown to play a key role in glioblastoma pathology, although its role in ferroptosis has not been directly investigated (105).

It has also been shown that the knockdown of RAB7A (member RAS oncogene family 7A) and autophagy-associated gene 5 (ATG5), a cargo receptor of lipid droplets, can prevent induced lipid peroxidation and subsequent ferroptosis (104, 106). Mechanistically, RAB7A is an indispensable factor for docking lysosomes to the lipid droplet surface during lipophagy under nutrient deprivation (107). Moreover, RAB7A has been demonstrated to act as a tumor suppressor in glioblastoma and prostate cancer (108), unlike other cancers.

Although emerging evidence reinforces the idea that the amount of lipid and the localization of lipids in lipid droplets potentially affect the ability to induce ferroptosis in cancer cells, studies on the effect of lipolysis and lipophagy on sensitizing glioblastoma cells for ferroptosis are limited. There is limited information on how microenvironment and disease progression affect the biogenesis and breakdown of lipid droplets (101). There is a need for a better understanding of specific trafficking itinerary of lipophagy which may lead to new anticancer approaches, particularly against “lipid addicted tumors” like glioblastoma. Finally, the molecular regulators of 1-O-acylceramide hydrolysis and whether this process impacts tumorigenesis are unknown.

## Summary

In the field of cancer metabolism reprogramming and plasticity, we have learned some fascinating lessons. One of the classical examples of metabolic reprogramming is the Warburg effect (109) which was reported almost 100 years ago and demonstrated a high glucose uptake and an increase in the glycolysis rate in cancer cells in an aerobic environment. In addition to high glucose consumption, glioblastoma has increased glutamine uptake rate to fuel proliferation compared with healthy cells. Emerging data have also shown that lipid metabolism provides an important energy substrate and carbon source for glioblastoma cells, affecting cancer cell plasticity and persistence during therapy (110, 111). Multiple lines of evidence also suggest that lipid droplets act as a central hub for lipid trafficking in glioblastoma, allowing lipids to move in and out of lipid droplets.

However, developing cancer treatments by targeting altered lipid metabolism is in its infancy. It remains challenging, primarily due to an incomplete understanding of the mechanisms that regulate lipid synthesis, esterification, lipolysis and lipophagy in cancer cells (34, 36, 58, 112, 113). In addition, our understanding of the pathways downstream of peroxidized phospholipids that execute ferroptosis remains extremely limited (**Box 1**). For instance, it is unclear whether peroxidized phospholipids are cytotoxic by themselves or when metabolized into products (e.g., electrophilic intermediates) that function as the primary drivers of ferroptosis or other adaptive metabolic reprogramming. The extent to which a physiological niche (cell/tissue function) alters lipid droplets contributions to ferroptosis and the unique context-dependent vulnerabilities that can be targeted in combinatorial approaches have also not been well explored. Moreover, metabolic byproducts accumulate in the tumor and its microenvironment, increasing the need for cancer cells to engage waste management and recycling pathways, as shown in the case of other metabolic byproducts such as acetate, ammonia, and free fatty acids (73–76, 114–116). However, the existence of waste management and the recycling of peroxidized phospholipids are not yet clear and require further study.

What is beyond question is the continued lack of life-prolonging treatment for glioblastoma patients despite significant advances in genetic and molecular characterization and almost twenty years since the introduction of temozolomide as the standard of care. This necessitates the discovery of nuanced avenues to understand glioblastoma formation to overcome therapeutic resistance. Ferroptosis and lipid metabolism offer some very early promise in this direction and are worthy of further exploration in this lethal cancer.

**BOX 1 Unanswered questions.**

Although monitoring the balance of PUFAs and MUFAs is known to predict the sensitivity of cells to ferroptosis, cellular sensing and the feedback mechanism that balances PUFAs/MUFAs remain to be investigated.

There is imputes to define the relationship between lipid droplets, fatty acid metabolism, and ferroptosis. However, key protein mediators of these biological processes remain to be discovered.

The most important factors for ferroptosis-dependent cell death are lipid peroxides that self-propagate along the plasma membrane and result in the accumulation of oxidatively damaged lipids. Our understanding of the downstream pathways of peroxidized phospholipids that execute ferroptosis and potentially contribute to tumorigenesis remains at a nascent phase. The question remains whether there is a repair or antagonism mechanism of peroxide lipids at damage loci in the cell membrane. Are oxidatively damaged membranes prone to recycling? Can this metabolic plasticity contribute to glioblastoma therapeutic resistance? How do these factors cooperate their functions in cancer microenvironments?

Many cancers, including glioblastoma, consist of both stem and nonstem cells; however, the degree to which these cells are resistant to ferroptosis remains mostly unknown.

## Author contributions

Conceptualization, AB-G. Writing-original draft preparation, AB-G, JD, and MW. Review and editing, AB-G, JD, and MW. Funding acquisition, AB-G. All authors have read and agreed to the published version of the manuscript.
